# Type I intrinsically photosensitive retinal ganglion cells of early post-natal development correspond to the M4 subtype

**DOI:** 10.1186/s13064-015-0042-x

**Published:** 2015-06-21

**Authors:** Timothy J. Sexton, Adam Bleckert, Maxwell H. Turner, Russell N. Van Gelder

**Affiliations:** Department of Ophthalmology, University of Washington, 325 9th Avenue, Seattle, WA 98104 USA; Department of Biological Structure, University of Washington, 1959 NE Pacific Street, Seattle, WA 98195 USA; Department of Physiology & Biophysics, University of Washington, 1705 NE Pacific St., Seattle, WA 98195 USA; Department of Pathology, University of Washington, 1959 NE Pacific Street, Seattle, WA 98195 USA; Program in Neurobiology and Behavior, University of Washington, Health Sciences Center, Seattle, WA 98195 USA

**Keywords:** Intrinsically photosensitive retinal ganglion cells, ipRGCs, Melanopsin, Adaptation, M4, Type 1

## Abstract

**Background:**

Intrinsically photosensitive retinal ganglion cells (ipRGCs) mediate circadian light entrainment and the pupillary light response in adult mice. In early development these cells mediate different processes, including negative phototaxis and the timing of retinal vascular development. To determine if ipRGC physiologic properties also change with development, we measured ipRGC cell density and light responses in wild-type mouse retinas at post-natal days 8, 15 and 30.

**Results:**

Melanopsin-positive cell density decreases by 17 % between post-natal days 8 and 15 and by 25 % between days 8 and 30. This decrease is due specifically to a decrease in cells co-labeled with a SMI-32, a marker for alpha-on ganglion cells (corresponding to adult morphologic type M4 ipRGCs). On multi-electrode array recordings, post-natal day 8 (P8) ipRGC light responses show more robust firing, reduced adaptation and more rapid recovery from short and extended light pulses than do the light responses of P15 and P30 ipRGCs. Three ipRGC subtypes – Types I-III – have been defined in early development based on sensitivity and latency on multielectrode array recordings. We find that Type I cells largely account for the unique physiologic properties of P8 ipRGCs. Type I cells have previously been shown to have relatively short latencies and high sensitivity. We now show that Type I cells show have rapid and robust recovery from long and short bright light exposures compared with Type II and III cells, suggesting differential light adaptation mechanisms between cell types. By P15, Type I ipRGCs are no longer detectable. Loose patch recordings of P8 M4 ipRGCs demonstrate Type I physiology.

**Conclusions:**

Type I ipRGCs are found only in early development. In addition to their previously described high sensitivity and rapid kinetics, these cells are uniquely resistant to adaptation and recover quickly and fully to short and prolonged light exposure. Type I ipRGCs correspond to the SMI-32 positive, M4 subtype and largely lose melanopsin expression in development. These cells constitute a unique morphologic and physiologic class of ipRGCs functioning early in postnatal development.

**Electronic supplementary material:**

The online version of this article (doi:10.1186/s13064-015-0042-x) contains supplementary material, which is available to authorized users.

## Background

Rods, cones, and melanopsin-containing intrinsically photosensitive retinal ganglion cells (ipRGC) account for all behaviorally relevant visual and non-visual photoreception in the murine retina [1]. Mice are born visually blind, with rods and cones not forming active synapses until approximately post-natal day 10 (P10) and eyes not opening until P13–15 [2–4]. In contrast, ipRGCs produce light induced spikes from birth (P0) [5–7]. Recent work suggests ipRGCs have unique roles during this early developmental period that are separate from their adult roles in circadian entrainment and the pupillary light reflex (PLR). These include melanopsin-dependent influences on the early post-natal development of retinal vasculature [8] and the segregation of retinogeniculate projections [9]. The latter is thought to be a consequence of ipRGC modulation of intrinsic retinal waves [9, 10]. Melanopsin also mediates negative phototactic behavior [11] and its accompanying vocalizations [12] in young pups.

Changes in ipRGC photosensitivity between early post-natal and adult ages have been reported in *rd1/rd1* mice [5] and in transgenic mice expressing eGFP under the melanopsin promoter [13]. However, these differences have not been systematically studied in large numbers of cells from wild-type mice. It remains unclear whether changes in ipRGC photosensitivity mirror the changes in ipRGC functional roles from birth to adulthood in wildtype animals. Additionally, while several physiologically distinct ipRGC subtypes have been described in the early post-natal mouse retina (Types I–III), these types have not been associated directly with the morphologic classes of ipRGCs (M1–M5) described in adults [5, 14]. Here we study wild-type ipRGC light responses over the course of post-natal development using multi-electrode array recording, and find a general reduction in photosensitivity with increasing age. This reduction in light sensitivity is largely restricted to one electrophysiologic subtype of ipRGC (the Type I cell). We also note a major reduction in the melanopsin expression in one anatomic subtype of ipRGC (the SMI-32**+**, M4 ipRGC) during post-natal development. Loose patch recordings confirm that these neonatal M4 cells possess Type I physiology. Mice thus possess a specific population of ipRGCs with heightened intrinsic photosensitivity in early development that is largely lost in adulthood.

## Results

### Changes in ipRGC and melanopsin expression in early development

**Fig. 1:**
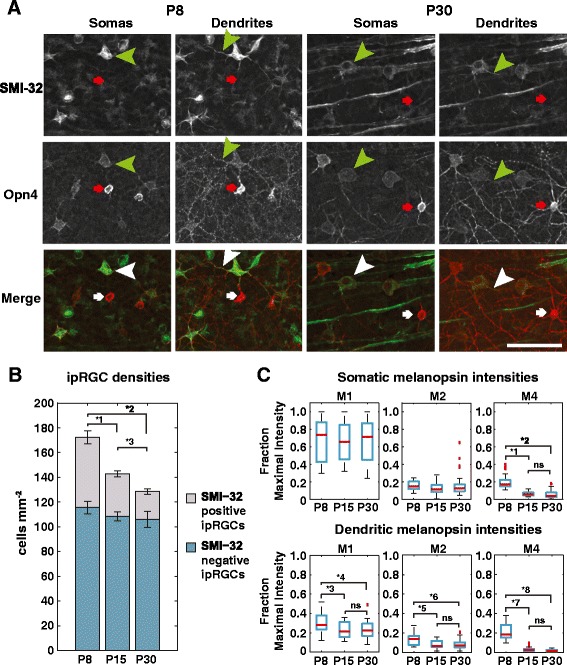
Melanopsin positive cell density decreases with age. **a** melanopsin and SMI-32 co-labeling in P8 and P30 retinas. Images emphasize either cell somata or cell dendrites. Arrowheads highlight co-labeled cells while line arrows highlight M1 cells. Scale bar = 50 μm. **b** Average densities of melanopsin-only positive cells (bottom bars) and melanopsin/SMI-32 co-labeled cells (top bars) in P8, P15 and P30 (*n* = 16 samples for each age). No significant difference in melanopsin**+**/SMI-32**−**. For melanopsin**+**/SMI-32**+** cells ANOVA, Bonferroni post hoc *1: *p* = 0.0003, *2: *p* = 7.8×10^−8^, *3: *p* = 0.056. **c** Relative pixel intensities for M1, M2 and M4 somata and dendrites at P8, P15 and P30. Data is ratio of a cell structure average pixel intensity to the average pixel intensity of the brightest cell soma measured in the same image. For each subtype/age category *n* = 24. *1: *p* = 9.6×10^−9^, *2: *p* = 1.0×10^−7^, *3: *p* = 0.058, *4: *p* = 0.050, *5: *p* = 0.009, *6: *p* = 0.002, *7: *p* = 6.9×10^−9^, *8: *p* = 8.4×10^−9^, K-W, followed M-W and B-c

During the large-scale apoptotic events of early retinal development ipRGCs numbers drop dramatically. Their numbers then stabilize before eye-opening and into adulthood [7, 15]. However, Tu et al. [5] showed a further decrease in the number of light active ipRGCs between P8 and adulthood. To study the change in ipRGC numbers during the post-apoptotic period, we measured ipRGC densities in wildtype P8, P15, P30 and P150 animals by melanopsin immunohistochemistry (see Fig. 1a). The density of total melanopsin-positive cells decreased by 17 % between P8 and P15 (from mean 173 mm^−2^ to 143 mm^−2^, *p* = 0.025 by ANOVA, Additional file 1 for details), 10 % between P15 and P30 (from 143 mm^−2^ to 129 mm^−2^, non-significant), and 25 % between P8 and P30 (173 mm^−2^ to 129 mm^−2^, *p* = 0.001) (Table 1, Fig. 1b). These densities are consistent with other recent ipRGC surveys [16, 17].

**Table 1:** Average ipRGC densities

Age	Total mel + mm^−2^	mel+/ SMI- mm^−2^	mel+/ SMI+ mm^−2^	Total SMI+ mm^−2^	% SMI+ cells also mel+	M1 mm^−2^	M2 mm^−2^	M3 mm^−2^	Displaced M1 mm^−2^
P8	173 ± 9	116 ± 9	57 ± 5	76 ± 6	76 ± 2	39 ± 2	74 ± 6	3 ± 1	13 ± 3
P15	143 ± 6	108 ± 4	35 ± 3	58 ± 5	63 ± 4	39 ± 2	68 ± 2	2.8 ± 0.4	11 ± 2
P30	129 ± 8	106 ± 6	23 ± 2	54 ± 4	43 ± 3	36 ± 2	67 ± 6	3 ± 1	10 ± 2
P150	56 ± 2	54 ± 2	1.1 ± 0.3	60 ± 8	3 ± 1	28 ± 2	31 ± 2	1.0 ± 0.3	8 ± 2

Average densities for melanopsin and SMI-32 stained cells

To determine whether this reduction was specific to ipRGC subtypes, counted cells were subtyped by morphology of immunohistochemically stained cells. The subtypes M1, M2 and M3 were characterized by dendritic arborization in the IPL off-layer, on-layer, or both, respectively. Estevez et al. (2012) [20] previously demonstrated that the ipRGC M4 subtype is also an alpha-on ganglion cell. The neurofilament marker SMI-32 is a marker for alpha-on ganglion cells [18]. Therefore, M4 cells were characterized by melanopsin and SMI-32 co-labeling coupled with dendritic projections to the IPL on-sublayer. Previously, M4 cells have been described only in adults [19–21]. Here, we extend that nomenclature to include melanopsin+/SMI-32+ cells in the retina from P8 animals. For analysis, cells were grouped into 2 categories: 1) melanopsin**+**/SMI-32**+** cells likely representing M4 cells, and 2) melanopsin**+**/SMI-32**−** cells representing M1, M2, and M3 subtypes (Table 2).

**Table 2:** Anatomical ipRGC subtypes

Anatomical subtype	Immuno-labeling	Dendritic projection/ ramification
M1	Melanopsin	Off-layer of IPL
M2	Melanopsin	On-layer of IPL
M3	Melanopsin	On- and Off-layer of IPL
M4	Melanopsin/SMI-32 co-labeling	On-layer of IPL

Method for categorizing ipRGC anatomical subtypes

Between P8 and P15, the melanopsin**+**/SMI-32**−** cell density decreased by 7 % (from 116 mm^−2^ to 108 mm^−2^, ns), while between P15 and P30 this density did not change (Table 1, Fig. 1b). As a group, the numbers of M1, M2 and M3 cells were stable across the ages studied. We also found that at P150 M2 density decreased by half (from 74 mm^−2^ to 31 mm^−2^) but M1 and M3 densities remained constant (Table 1). In contrast, melanopsin**+**/SMI-32**+** cell density decreased 38 % between P8 and P15 (from 57 mm^−2^ to 35 mm^−2^*p* = 2.8x10^−4^ ANOVA, Additional file 1), 34 % between P15 and P30 (from 35 mm^−2^ to 23 mm^-2,^*p* = 0.056), and 60 % between P8 and P30 (from 57 mm^−2^ to 23 mm^−2^, *p* = 7.8×10^−8^). Therefore, the decrease in melanopsin**+**/SMI-32**+** (presumed M4) cell density account for nearly all of the decrease in total ipRGC density.

**Fig. 2:**
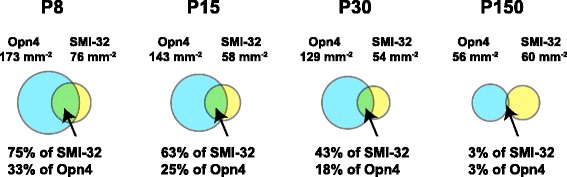
Melanopsin+/SMI+ cell density decreases with age. Venn Diagrams of melanopsin**+**/SMI**+** cell distribution among all melanopsin and all SMI-32 positive cells at each developmental age studied

This decrease arises from two processes. First, the density of total SMI-32**+** cells (regardless of melanopsin staining) decreased with age: 24 % from P8 to P15 (from 76 mm^−2^ to 58 mm^−2^*p* = 0.061, ANOVA, Additional file 1), 7 % from P15 to P30 (from 58 mm^−2^ to 54 mm^−2^, non-significant) and 29 % from P8 to P30 (from 76 mm^−2^ to 54 mm^−2^, *p* =0.015) (Table 1). Second, the average percentage of SMI-32**+** cells that were also melanopsin **+** decreased with age: from 76 ± 2 % at P8, to 63 ± 4 % at P15, and to 43 ± 4 % at P30 (ANOVA, Additional file 1, all *p* < 0.05). By P150 this percentage dropped to 3 ± 1 % (1.1 ± 0.3 cell mm^−2^, *n* = 3 retinas) (Table 1, Fig. 2). These results indicate that the decrease in melanopsin**+**/SMI-32**+** cells (M4) is from 1) a small decrease in SMI-32**+** cell density, possibly from retinal growth or residual apoptosis, and 2) a marked decrease in the percentage of remaining SMI-32**+** cells expressing detectable melanopsin.

We measured melanopsin expression levels in somata and dendrites of each subtype by calculating relative pixel intensities in confocal images. The average pixel intensities of somatic and dendritic melanopsin staining were measured from each of 6 M1, M2 and M4 cells within a single image. A total of 4 images from different retinas were analyzed at each age for a total of 24 cells per subtype, per age. Within each image all measurements were normalized to the intensity of the brightest cell measured (always an M1 cell) and termed relative intensities. The median relative intensities for M1 somata remained stable across ages regardless of absolute melanopsin levels. M1 dendritic intensities had a non-significant trend toward lower expression (Fig. 1c). Median relative intensities for M2 somata were also stable across development, remaining at ~30 % of M1 values. However, median dendritic intensities for M2 cells decreased by 50 % between P8 and older retinas (*p* <0.05, Kruskal-Wallis (K-W), Additional file 1). Median relative intensities for M4 somata dropped by 30 % between P8 and older retinas (*p* < 0.005, K-W, Additional file 1), while dendritic values decreased 8 fold over the same time (*p* < 0.005, K-W, Additional file 1) (Fig. 1c).

### Light response dynamics of ipRGCs in early development

The pronounced loss of melanopsin expression in M4 cells and the more moderate loss in M1 and M2 cells could result in altered intrinsic photosensitivity over the course of development. Changes in ipRGC photosensitivity between birth and adulthood have been noted previously in retinas of *rd1/rd1* animals [5], and in transgenic animals expressing eGFP under control of the melanopsin gene locus [13]. To test for altered photosensitivity in wildtype animals, we assessed ipRGC light responses at P8, P15 and P30 using multielectrode array recordings.

With increasing age, the number of ipRGCs recordable by MEA decreased: 101 cells were recordable from 5 P8 retinas (average = 20.2 cells per retina), 35 cells from 14 P15 retinas (2.5 cells per retina), and 18 cells from 9 P30 retinas (2.0 cells per retina). For P15 and P30, these averages exclude retinas with no recordable ipRGCs, which constituted 50 % and 30 % of P15 and P30 retinas respectively. Including all retinas, the average ipRGC per retina in P15 and P30 animals is close to 1.

**Fig. 3:**
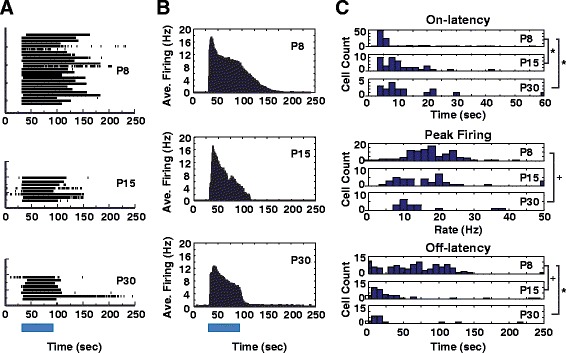
With age ipRGCs become fewer, show slower firing rate, slower on-latency, and faster off-latency. **a** Raster plots of representative ipRGCs from P8 (*n* = 25), P15 (*n* = 8), P30 (*n* = 8). **b** Histograms of average ipRGC firing from cells depicted in A. **c** Histograms of P8 (*n* = 101), P15 (*n* = 35) and P30 (*n* = 18) light response dynamics including on-latency, peak firing and off-latency. *: *p* < 0.05, K-W, followed M-W and B-c

Spiking light responses were recorded following a 1-min 480 nm light stimulus at 3.98 × 10^13^ photon cm^−2^ s^−1^ (IR 13.6) (Table 3). Median on-latency increased from a mean of 6 s post lights-on at P8 to 9.5 s at P15 and 9 s at P30 (*p* < 0.05, K-W, Additional file 2). Median off-latency decreased significantly between the same groups, from 67 s at P8 to 17.5 s at P15 and 15.5 s at P30 (*p* < 0.05, K-W, Additional file 2). Peak firing rate decreased significantly between P8 and P30 only (*p* = 0.003, K-W, Additional file 2) (Table 3, Fig. 3). As a consequence of these changes, the total number of spikes elicited by a 1-min stimulus decreased significantly with age, from median 966 spikes at P8 to 410 spikes at P30 to the same stimulus (*p* < 0.05, K-W, Additional file 2). Overall these changes demonstrate an aggregate decrease in photosensitivity, consistent with other studies [5, 13].

**Table 3:** Age specific light response parameters

	P8	P15	P30
On-latency (sec)	6, 5–18	9.5, 6–15	9, 6–20
Off-latency (sec)	67, 31–99.5	17.5, 8–37	15.5, 6–20
Peak firing (Hz)	18, 13.5–23	16, 9–21	11.5, 10–15
Total Spikes	966, 566–1273	630, 430–851	410, 294–646

Summary of age specific light response parameters. Reported as median value and interquartile range

### Recovery from 1-min bright light exposure

**Fig. 4:**
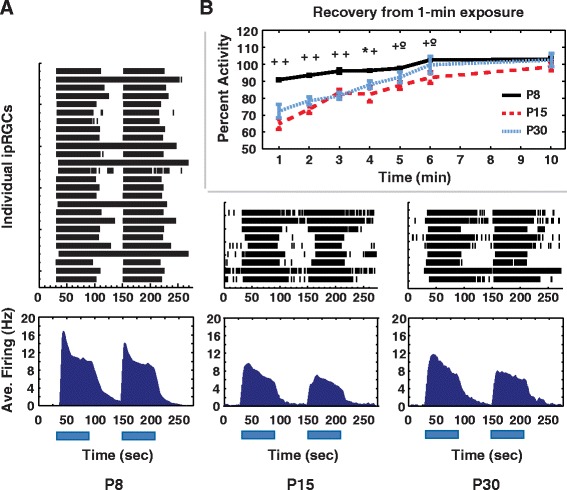
ipRGCs of younger mice recover more quickly following a short light exposure than ipRGCs or older mice. **a** Rasters and average histograms of representative ipRGCs used in time course experiments **b** Time courses of P8 (*n* = 74), P15 (*n* = 10 to 15), and P30 (*n* = 6 to 16) ipRGC recovery following a 1-min light exposure. *: *p* < 0.05, **+**: *p* < 0.005, **°:** ns. For each time point, first significance symbol is for P8-P15 comparisons and second symbol is for P8-P30 comparisons. LMM, followed by one-way ANOVA and Bonferroni post-hoc for each time point

Adult ipRGCs are remarkably resistant to bleaching [22] and show slow adaptation, with some cells continuing to fire *ex vivo* after many hours of continuous light exposure [23]. The adaptation and bleaching kinetics of developing ipRGCs have not been previously studied. We first tested the ability of developing ipRGCs to recover from a 1-min, 480 nm light at IR 13.6. Exposed retinas were allowed to recover for 1 to 10-min in the dark, and then retested with the same light stimulus. Recovery in each test stimulus was calculated as ratio of spikes during the test stimulus compared to the first 1-min light. P8 ipRGCs recovered to 90 % after a 1-min recovery period and recovered completely by 6 min. Cells from P15 and P30 animals recovered to only 60−70 % after 1-min recovery and required more than 6 min to fully recover (Fig. 4b). The differences in recovery times between P8 and P15 ipRGCs and P8 and P30 ipRGCS were statistically significant up to the 4-min recovery point. At 5-min and 6-min only the difference between P8 and P15 was significant (*p* < 0.05, Fig. 4b; LMM, Additional file 3 for details). Changes in peak firing rate and off-latency in recovery mirrored those of total spike activity. On-latencies for P8 cells remained near 100 % of pre-exposure values for all test intervals. On-latencies of P15 and P30 cells initially increased to a mean of 150 % pre-exposure values (longer latencies), but returned to 100 % by10-min recovery.

During 1-min recovery experiments, ipRGCs from P15 and P30 animals ‘fatigued’ following multiple 1-min light exposures, recovering to between only 40 and 60 % of baseline after 3 to 4 recovery trials, regardless of interval length. This was true even with a 15-min dark period between interval tests. P8 cells did not ‘fatigue.’ Thus, we utilized only data from cells exposed to 2 or fewer recovery cycles. This result does suggest that the recovery mechanisms of P8 ipRGCs differ substantially from those of older animals.

### Recovery from 1-h bright light exposure

**Fig. 5:**
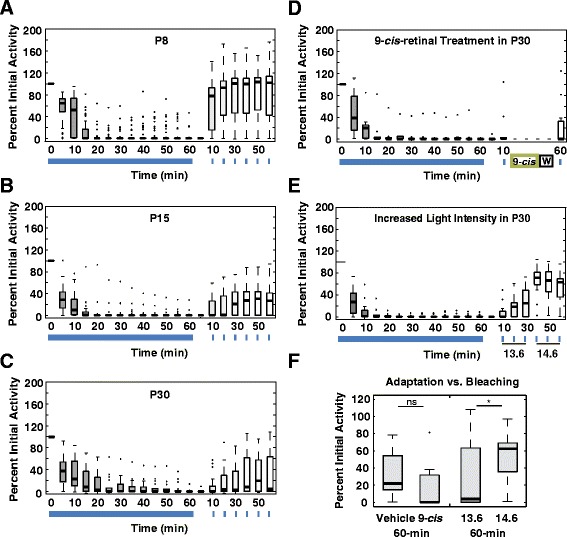
Older ipRGCs show increased adaptation in response to a 1-h bright light stimulus. Boxplots of exposure and recovery time courses for (**a**) P8 (*n* = 60), (**b**) P15 (*n* = 23), and (**c**) P30 (*n* = 17). Boxplots of 1-h exposure followed (**d**) 9-*cis*-retinal treatment (100 μM) or (**e**) a 10-fold higher light intensity during the recovery period. **f** Comparison of 9-cis-retinal treatment with vehicle control (1 % ethanol in AMES) and the 10-fold higher light level (IR 14.6) with the lower light level (IR 13.6). *: *p* = 0.01, M-W

ipRGCs are noted for their ability to respond to long light exposures [22–24]. To determine how cell firing and recovery from long light exposures varies with development, we next exposed retinas of P8, P15, and P30 mice to a 1-h light (480 nm, IR 13.6), and followed recovery for 1-h with a 1-min test light (same as exposure light) every 10-min (Fig. 5a–c). To test cell health, retinas were treated with KCl at the conclusion of each experiment. During the 1-h exposure, total spiking decreased over the first 20-min and stopped by 30-min in most cells. A small number of cells continued to fire throughout the 1-h. This pattern was seen at all ages. During recovery, age-specific differences were observed. Recovery was measured as a ratio of the total spikes during the 1 min post-exposure pulse compared with the first minute of the 1-h light exposure for each cell. Total spikes in P8 retinas recovered quickly, reaching 100 % by 20-min of dark recovery after the 1-h exposure. In comparison, P15 and P30 cells recovered much more slowly, reaching medians of 30 % and 20 %, respectively, over 1 h (Fig. 5a-c and Additional file 1). Between P8 and older ages there were also significant decreases in recovery of peak firing rate, on-latency, initial spikes (first 30-s of firing) and steady state spikes (second 30-s of firing) (all *p* < 0.05, LMM, Additional file 4: Figure; Additional file 5). Interestingly, off-latencies showed the opposite pattern. P15 and P30 cells recovered off-latencies near that of pre-exposure levels, while P8 cells never recovered to pre-exposure off-latencies (*p* = 0.039; LMM,; Additional file 4: Figure; Additional file 5).

### Bleaching vs. adaptation

The observed age-dependent decreases in recovery after a 1-h light exposure could be caused by changes in either susceptibility to bleaching or to changes in light adaptation mechanisms (or both). To distinguish between these two possibilities, ipRGCs from P30 animals were exposed to either 9-*cis*-retinal to reverse bleaching, or to a ten-fold higher intensity light to test for adaptation. Using a protocol that allows retinaldehyde to penetrate the retina on an MEA and restore bleached ipRGC function [24], treatment with 9-*cis*-retinal during the recovery period did not increase recovery levels above vehicle control or untreated cells (Fig. 5d). Increasing light intensity at the 40, 50 and 60-min recovery time points increased percent recovery over control at all three times points (*p* = 0.01, Mann-Whitney, 60-min time point; Fig. 5f). Therefore the attenuated responses after long light exposure were not due to irreversible ‘run down’ in the tissue preparation or chromophore depletion. Taken together, these results suggest that changes in adaptation rather than melanopsin bleaching are responsible for age-dependent changes in recovery.

### Recovery of ipRGC subtypes from bright light exposure

The age specific changes in adaptation can be further understood by looking at changes in ipRGC subtypes. Based on MEA recordings, Tu et al. (2005) defined three distinct ipRGC subtypes – denoted Type I, II, and III – based on photosensitivity and on-latency. Type I cells show relatively high sensitivity but slow on-latency; Type II cells show relatively low sensitivity and slow on-latency, and Type III cells show both relatively high sensitivity and relatively fast on-latency. We sought to determine if these subtypes showed differences in adaptation to long light exposure and how these differences might change in early development.

**Fig. 6:**
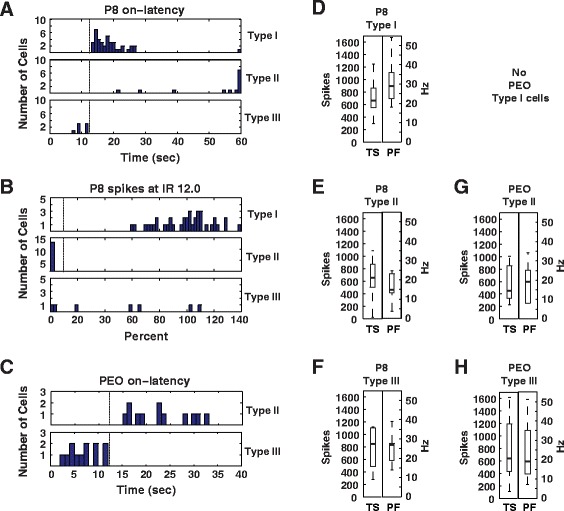
ipRGCs are subtyped by photosensitivity and on-latency. Panel 1 (**a**) contains histograms of classifications parameters include (**a**) P8 cell on-latency at IR 12.0 – dashed line indicates the 12-s selection criterion, (**b**) P8 cell percentage of spikes at IR12.0 – dashed line indicates the 10 % selection criterion between Types I and II, (**c**) Post-eye opening cell (PEO) on-latencies – dashed line indicates the 12-s selection criterion. Panel 2 contains boxplots for the response characteristics of total spikes (TS) and peak firing (PF) from the first minute of the one hour light exposure (**d**) P8 Type I (*n* = 40), (**e**) P8 Type II (*n* = 13), (**f**) P8 Type III (*n* = 7), (**g**) Post-eye-opening Type II (*n* = 13), (**h**) Post-eye-opening Type III (*n* = 12)

Cells were subtyped as in Tu et al. (2005) with small modifications to reduce total light exposure before experiments (Table 4, Fig. 6a–c). Because of the relative paucity of total recordable Type II (*n* = 13) and III (*n* = 12) cells in older animals, P15 P30 subtypes were combined into a single ‘post-eye-opening’ group. This combination was justified by the lack of statistically significant differences in P15 and P30 responses (Figure: Additional file 4). Total spikes and peak firing from the first minute of the 1-h light exposure showed a range of healthy responses in each subtype consistent with their relative photosensitivity (Fig. 6d–h). All subtypes had similar responses to KCl treatment at the end of each experiment and were assumed to be of similar health (Table 5). Importantly, median KCl responses did not predict subtype recovery levels.

**Table 4:** Electrophysiologic ipRGC subtypes

Age	Type I	Type II	Type III
P8	On-latency > 12-s at IR 12.0	On-latency > 12-s at IR 12.0	On-latency ≤ 12-s at IR 12.0
	Light response at IR 13.6 with response at 12.0 of > 10 % 13.6 response	Light response at IR 13.6 with response at 12.0 of ≤ 10 % 13.6 response	
P15 and P30	Not present	On-latency > 12-s at IR 13	On-latency ≤ 12-s at IR 13

Method for categorizing ipRGC electrophysiologic subtypes. Irradiance (IR) is presented as the log_10_ (photon cm^−2^ s^−1^). On-latency is the time from lights on until peak firing rate

**Table 5:** Responses of ipRGCs to excess KCl

	P8 Type I	P8 Type II	P8 Type III	PEO Type I	PEO Type II
Median	64 %	50 %	51 %	84 %	36 %
Interquartile Range	43–169 %	28–153 %	24–121 %	43–159 %	10–115 %

KCl induced firing of P8 and post-eye-opening (PEO) cells ipRGCs reported as a percentage of cell firing during the first minute of the 1-h light exposure. Cells with less than a 10 % KCl response were not analyzed

**Fig. 7:**
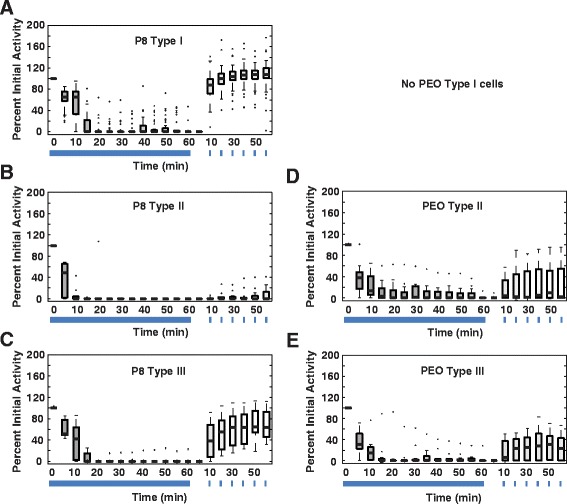
P8 ipRGC recovery following bright light exposure is dominated by Type I cells. Boxplots of exposure and recovery time courses for a subset of cells from the ages in Fig. 5a-c. **a** P8 Type I (*n* = 40), (**b**) P8 Type II (*n* = 13), (**c**) P8 Type III (*n* = 7), (**d**) Post-eye-opening Type II (*n* = 13), (**e**) Post-eye-opening Type III (*n* = 12)

In P8 retinas the three ipRGC subtypes responded to and recovered from long, bright light exposure with significantly different dynamics. During the 1-h exposure, the vast majority of cells responded to light for the first 20-min. A subset of Type I cells continued firing throughout the 1-h light exposure either non-stop (4 cells, 9 %) or intermittently (5 cells, or 11 %). In contrast, 1 out of 7 Type III cells continued to fire continuously during the 1-h exposure, while none of the 13 Type II cells did (Fig. 7, Table 6). Following the 1-h exposure, Type I cells recovered more fully than Type III cells, and Type III cells recovered more fully than Type II cells. In Type I cells total spikes recovered to a median of just over 100 % pre-exposure activity. In Type III cells total spikes recovered to a median of 70 % of pre-exposure activity. In contrast, Type II cells showed minimal recovery after 1-h (Fig. 7 and Additional file 6; *p* < 0.05 for differences between subtype LMM, Additional file 7). There were also significant subtype differences in on-latency, peak-firing, and off-latency, initial spikes and steady-state spikes (all *p* < 0.05, LMM, Additional file 7, Additional file 6: Figure)

**Table 6:** Summary of age-dependent ipRGC changes

Parameter	P8 Type I	P8 Type II	P8 Type III	P15/30 Type II	P15/30 Type III
Percent cells with continuous firing during exposure	9 %	0 %	0 %	23 %	16 %
Percent cells with intermittent firing during exposure	10 %	0 %	0 %	23 %	15 %
Recovery values: On-latency (sec)	11	61	12	33	26
Recovery values: Off-latency (sec)	27	11	27	26	7
Percent recovery: Peak Firing	65 %	0 %	55 %	25 %	13 %
Percent recovery: Total Spikes	108 %	0 %	40 %	3 %	27 %
Percent recovery: Initial Spikes	92 %	0 %	65 %	2 %	16 %
Percent recovery: Steady-state Spikes	138 %	1 %	76 %	3 %	33 %
Anatomically corresponding cell	M4	M2	M1	M2	M1
Somatic melanopsin level in anatomically corresponding cell over development	**↓**	No significant change	No significant change		
Dendritic melanopsin level in anatomically correspondent cell over development	**↓↓**	**↓**	**↓**		

Summary of changes in light exposure parameters, recovery parameters, and melanopsin expression levels in cell subtypes over development. Percentages are greatest median percent of pre-exposure values recovered over the 1-h recovery. Time measurements are the greatest median times recovered over the 1-h. Post-eye-opening (PEO)

Post-eye-opening Type II and Type III cells did not differ significantly from each other in any light response parameters measured. Within each subtype, however, differences in response and recovery were observed with age. Both post-eye-opening Type II and III cells showed a greater proportion of cells firing intermittently or continuously than the same class of cells at P8 during the 1-h exposure (Fig. 7, Table 6). This suggests that a subset of both cell types exhibited reduced adaptation to continuous exposure with increasing age. Type II cells showed an increase in recovery (decreased adaptation) with age in on-latency, total spikes, and initial spikes. In contrast, Type III cells showed a significant decrease in recovery (increased adaptation) with age in peak firing, total spikes, and initial spikes (LMM, Additional file 8, all *p* < 0.05; Additional file 6: Figure).

### Patch clamp recordings of M4 ipRGCs show type I physiology exclusively

**Fig. 8:**
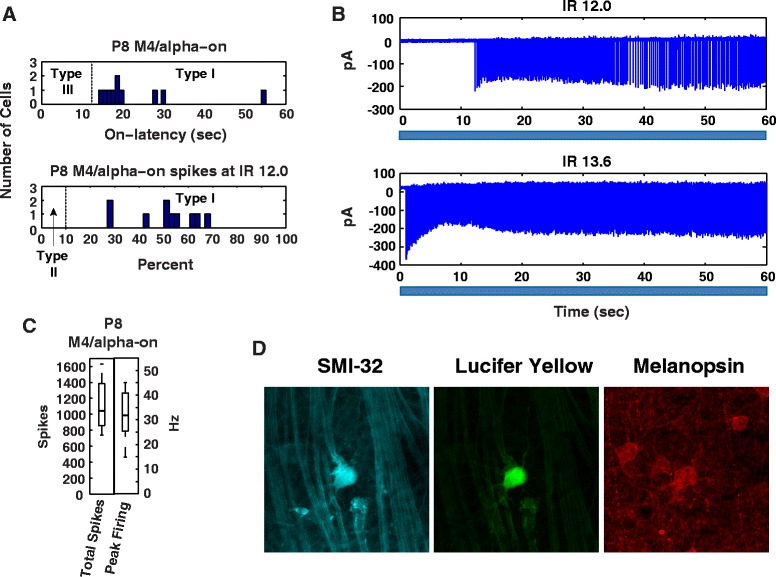
M4/alpha-on cells have Type I cell physiology. **a** M4 light responses fall into the Type I category using the MEA subtyping criteria for on-latency and photosensitivity. **b** Loose patch traces of M4 cell at IR 12.0 and 13.6. **c** M4 cells have a range of responses in total spiking and peak firing similar to MEA Type I cells. **d** The identity of loose patch recorded cells was confirmed by co-staining with lucifer yellow, melanopsin and SMI-32 antibodies. Scale bar = 50 μm

By P15, Type I cells were not detectable. Since M4/alpha-on cells showed a decreased melanopsin expression over the same time course, we tested P8 M4 cells with loose patch recordings to determine their electrophysiologic subtype (Types 1–III). In wildtype P8 retina recordings were made from large cells with alpha-on appearance, which were later confirmed to be M4/alpha-on cells by SMI-32 and melanopsin co-staining (Fig. 8d). Using the MEA subtyping criteria (Table 4) 10/10 patched cells were identified as Type I cells (Fig. 8a).

## Discussion

Recent work has demonstrated that ipRGCs subserve different aspects of light-dependent behavior and physiology in neonatal mice compared with adults [8, 9, 11, 12]. Here we demonstrate that ipRGCs themselves undergo marked physiologic changes between P8 and P30. We find the previously described decrease in total ipRGC number after P8 [5, 7] is specifically due to loss of melanopsin expression in SMI-32**+**, alpha-on ganglion cells, which in the adult correspond to the M4 subclass of ipRGCs [19, 21]. As the density of M1 and M2 cells does not appear to substantially decline during the same time period, it is unlikely the reduced M4-type cell density is due to generalized retinal growth. As electrophysiologically-defined Type I cells also disappear with the same time course, we hypothesized that these M4-type cells correspond to the Type I electrophysiologic class. Loose patch recording of M4/alpha-on cells confirmed they are Type I cells. This result rounds out the suggestions of Schmidt and Kofuji that Type II cells correspond to M2 cells, and Type III cells to M1 cells [25]. Additionally, we find that Type I cells have unique physiology and show remarkable recovery to long light exposure compared with early Type II or III cells. Conversely, we see little reduction in numbers of electrophysiologic Type II and III cells, but do see maturation in adaptation responses following long light exposure with development, suggesting these cells are maturing to subserve different functions in the adult animal. This includes more type II and III cells firing continuously in response to light as would be necessary for the PLR or for circadian entrainment. These results suggest that neonatal Type I/M4 cells are capable of subserving unique melanopsin-dependent functions in early development. This capacity decreases with age as melanopsin expression levels in these cells decrease.

The loss of Type I cells from MEA recordings is consistent with the 8-fold decrease in M4 melanopsin levels after P8. It is also consistent with patch-clamp recordings of adult M4s demonstrating a light response that is both smaller in photocurrent and 1 to 2 logs less photosensitive than M2 responses (Ecker et al. 2010). While M4 cells appear to be present at all ages, the low melanopsin expression in older M4 cells likely renders them undetectable in MEA recordings in the present study. A 2-log reduction in response threshold would place the adult M4 response beyond the intensity of the Xenon light source used here. Overall, this suggests that whereas the light response of P8 Type1/ M4 cells are driven entirely by melanopsin, in adults their light response is driven primarily by rod and cone input.

While 5 anatomically defined ipRGC subtypes have been described [26, 27], we did not look specifically at M5 cells because they lack specific markers. Additionally, there is debate whether or not these cells are a distinct subtype or a variant of M4 cells (both appear to be alpha-on cells [18]).

Previous studies have shown that adult M4 ipRGCs do not express melanopsin levels highly enough to be detected reliably by standard immunochemistry [20, 21, 28]. However, we find that in young post-eye-opening animals at least a subset of alpha-on cells, as labeled with SMI-32, do stain for melanopsin using standard immunohistochemistry. The difference in results between previous studies and this study most likely stem from the age of animals examined. Previous studies used P42–P120 animals while the current study used primarily P8 to P35 animals. Our finding that only 3 % of P150 M4/alpha-on cells express melanopsin suggests that as animals mature from post-eye-opening to adulthood (between P35 and P120), melanopsin levels become undetectable by standard techniques in all but a fraction of cells.

The decrease in Type I/M4 ipRGCs alone does not fully account for the decrease in recordable ipRGCs on multi-electrode recording seen with increasing age. While a decrease in total density from 173 mm^−2^ to 129 mm^−2^ was observed for melanopsin-positive cells between P8 and P30 (nearly all attributable to loss of melanopsin staining in SMI-32+ cells), the number of recordable cells per retina decreased from ~20 cells/retina to 2 cells per retina. The reason(s) for this further decrease are not apparent, but could include possibilities of decreased overall cell viability *in vitro* with increased age, or of melanopsin-positive cells that are non-spiking.

### Age-specific ipRGC light response recovery

As animals age, ipRGCs exhibit lower sensitivity to light. This is seen in the changes to cell on-latency, peak firing rate, off-latency, and total spikes. These changes are consistent with the results of Tu et al. (2005) and Schmidt et al. (2008). Additionally, the ability of ipRGCs to recovery from a 1-min light exposure decreases with age. Cells from older animals require nearly twice the time to fully recover than P8 cells, similar to recovery times for ipRGCs in older animals as reported by Do and Yau [29]. It is unlikely that the age-dependent decreases in light responses reflects melanopsin bleaching in the 1-min light exposures as mounting evidence indicates melanopsin is a bistable pigment [30] and highly resistant to photic bleaching [24].

Changes in recovery following a 1-h light exposure were more dramatic (Table 6). P8 Type I cells recovered to 100 % of initial activity after 20-min recovery while P15 and P30 cells recovered to only 20–40 % by the end of the 1-h recovery period. This raises the possibility that chromophore recycling and availability change with age, perhaps as a consequence of fully functioning rods and cones acting as sinks for available 11-*cis*-retinal in older animals. The absence of increased sensitivity after 9-*cis*-retinal supplementation argues against this. Instead, a difference in long-acting adaptation mechanisms is likely to explain these age-dependent changes, as suggested by increased light responses from older ipRGCs upon exposure to 10-fold brighter light. Such a prolonged recovery time is consistent with the work of Wong et al., [31] who showed, in older animals, that ipRGCs can take 3-h to dark adapt after even brief, strong light exposures.

Interestingly, changes in firing rate and off-latency following long light exposure appears to be decoupled. In P8 ipRGCs, the steady-state spike number recovered to greater than pre-exposure levels while off-latency never recovered to pre-exposure levels. In contrast, older ipRGC steady-state spiking never fully recovered but off-latency did. This suggests that mechanisms governing adaptation of steady state firing and signal termination are independent and differentially regulated with development.

Previous work by Wong et al. [31] demonstrated ipRGC adaptation responses that are similar to those in visual photoreceptors. Here we extend that work to adaptation of the functional output of ipRGCs (i.e. cell spiking) instead of direct measurements of photocurrents. This is an important measurement, as the ultimate effect of light exposure history must be reflected in signaling to targets like the suprachiasmatic nuclei (controlling circadian rhythms) and the olivary pretectum (controlling pupillary light responses) in adults. Our data demonstrate that functional adaptation in most ipRGCs is profound and long lasting. However, those cells that continued to fire throughout the 1-h light exposure showed relatively low levels of adaptation and high rates of recovery to pre-exposure values. These cells could correspond to the rat ipRGCs found by Wong exhibiting continuous firing in response to a 10-h light stimulus [23]. The major difference between this study and Wong is the use of a retinal pigment epithelium (RPE)-attached preparation in the latter. It would be of interest to see if a similar RPE attached preparation in mouse could decrease the adaptation of cells seen here, even when direct retinoid supplementation could not. Alternatively, the difference may stem from the difference in animal pre-experiment dark adaptation time. We dark adapted for 1-h prior to experiments, while Wong dark adapted for 15–20 h. If this is the case, then the difference in results of the two studies reflects a profoundly long-lasting adaptation mechanism in ipRGCs. The identity of the cells showing minimal adaptation is unclear. Both P8 Type I and post-eye-opening Type II and III populations had adaptation-resistant cells. Whether these represent one end of a distribution of adaptation, or represent a distinct subpopulation of cells (such as the Brn3b-positive or -negative M1 population [15]) remains to be determined.

## Conclusions

We find that there are general decreases in ipRGC density and photosensitivity with age and that these changes are subtype-specific, and primarily attributable to loss of Type I cells. Most importantly, we find that P8 Type I cells are M4/alpha-on cells and that loss of melanopsin in these cells likely accounts for the majority of changes observed. At P8 these cells behave differently from other subtypes, with increased sensitivity and reduced adaptation. This may reflect a simple maturation of Type1/M4 cells with no functional correlate. However, it is possible that the relative lack of adaptation in these cells could subserve developmental functions such as light modulation of spontaneous retinal waves in neonates and survival behaviors in post-natal animals like negative phototaxis in which sustained adaptation might be detrimental to the animal.

## Methods

### Mice

All experiments were performed in accordance with Association for Research in Vision and Ophthalmology guidelines for animal studies and under an approved animal study protocol at the University of Washington Institutional (Animal Care and Use Committee of University of Washington Protocol #4184-01). Mice were C57BL/6 (Jackson Laboratories, Bar Harbor, ME) studied at ages P8–P10 (P8), P15–20 (P15), and P30–P35 (P30). Animals were maintained in a 12:12-h light–dark cycle and provided food *ad libitum*.

### Immunohistochemistry and analysis

Melanopsin immuno-positive cell densities were determined at P8, P15, and P30. Morphologic ipRGC subtypes were identified by a combination of dendritic projection patterns and immunohistochemical markers using confocal microscopy (Table 2) [28, 32]. Melanopsin-immunopositive cells with dendritic projection to the off-sublayer of the inner-plexiform layer (IPL) were designated M1 cells, while those with dendritic projections to the on-sublayer of the IPL were designated M2/M4. Since M4 cells are also alpha-on cells (Estevez et al. [20]) M4 cells were differentiated from M2 cells by co-staining with melanopsin and the alpha-on marker non-phosphorylated neurofilament h (SMI-32) [18, 33–35]. For melanopsin and SMI-32 immunohistochemistry, retinas were fixed for 1-h in 4 % paraformaldehyde and blocked overnight in 5 % donkey serum, 2 mg/ml BSA, 0.3 % Tx-100 in PBS. Retinas were then co-incubated with both primary antibodies for 3 nights at 4 **°** C, using a custom polyclonal rabbit anti-mouse melanopsin antibody targeting the first 15 amino acids of melanopsin N-terminus ([1:5000]) and a mouse monoclonal antibody for SMI-32 ([1:1000], NE1023, Calbiochem, San Diego, CA). The melanopsin antibody used was previously characterized in [5], and does not stain retinas from melanopsin-deficient mice (data not shown). Secondary antibodies were incubated overnight at 4 **°**C. These were donkey anti-rabbit Alexa 568 (A10042, Invitrogen, Grand Island, NY) and donkey anti-mouse Alexa 488 (A21020, Invitrogen) for melanopsin and SMI-32 respectively. Retinas were imaged by confocal microscopy. A region (average area = 1.29 mm^2^) was sampled from each of the four retinal quadrants, with each region centered 1 mm from the optic disc. Four images per quadrant were combined into a single image. Cell counts were done by hand using the Cell Counter plug-in of Image J (NIH). Only cells with distinct somatic perimeter staining were counted as melanopsin positive. Counts were analyzed per group with a non-parametric Kruskal-Wallis test followed by a Mann-Whitney (2-tailed) test.

To determine the level of melanopsin staining in cell subtypes over time, relative average intensities were calculated for ipRGC somatic perimeters and dendrites of M1, M2 and M4 subtypes from P8, P15 and P30 mice. Average pixel intensity measurements were taken from structures in a single optical plane of confocal stacks with Image J (NIH). Measurements of soma perimeter average intensity were taken at each cell’s widest extent before dendritic projections appeared. M1 dendritic average intensities were measured and averaged from 3 dendrites measured from the distance a dendrite remained in the optical plan after its first ramification in the IPL off-sublayer. Dendritic intensities for M2 and M4 cells were measured from their emergence from the cell soma to the length they could be followed reliably, (usually 1 to 2 cell diameter lengths). Intensities were averaged for 2 and 3 dendrites per cell for M2 and M4 cells, respectively. All measurements were background-subtracted from an equivalent area adjacent to the structure measured. Six cells of each subtype were measured from a single image. All measured average intensity values for cell soma and dendrites were normalized to the value of the soma of the brightest cell measured per image (always an M1 cell). One image from each of 4 retinas was examined yielding a total of 24 cells per subtype per age. Intensity data was not normally distributed (Shapiro-Wilk test, *p* < 0.05) and did not have equal variance within cells types across age (Levene’s test, *p* < 0.05). Each cell type was therefore compared over the three ages with Kruskal-Wallis tests. Significant Kruskal-Wallis tests were followed by Mann-Whitney (2-tailed) tests and Bonferroni corrected for 3 comparisons for specific age differences. Statistical analysis was performed with SPSS (IBM, Armonk NY).

### Multi-electrode array (MEA) recordings

Mice were dark adapted for 1-h prior to experiments. Subsequent manipulations were performed under dim red light illumination. Mice were euthanized by CO_2_ narcosis and cervical dislocation. Retinas were isolated in bicarbonate-buffered physiologic solution (125 mM NaCl, 2.5 mM KCl, 1 mM MgCl_2_, 1.25 mM NaH_2_PO_4_, 20 mM glucose, 26 mM NaHCO_3_, 2 mM CaCl_2_, 500 μM glutamine) oxygenated with 95 % O_2_/5 % CO_2_ to obtain a pH of 7.4. Isolated retinas were cut in half, mounted on filter paper, positioned with the vitreal face in contact with a MEA (Multi Channel Systems, Reutlingen, Germany) and superperfused at 2–3 ml/min with a bicarbonate-buffered physiologic solution. The temperature of both perfusate and tissue chamber was maintained at 33.0 °C. For ipRGC recordings from P8 mouse retinas, spontaneous retinal waves were suppressed with a cholinergic inhibitor (5 nM epibatidine) [36]. Any possible input from rod and cone photoreceptors were suppressed with glutamatergic inhibitors [50 μM d(2)-2-amino-5-phosphonopentanoic acid (d-AP5); 20 μM d(−)-2-amino-4-phosphonobutyric acid (d-AP4), and 10 μM 6,7-dinitroquinoxaline-2,3-dione (DNQX)] (Tocris Biosciences, Ellisville, MO). For ipRGC recordings from P15 and P30 mouse retinas, as spontaneous wave activity had ceased, only glutamatergic blockade was used (200 μM d-AP5, 100 μM d-AP4, and 80 μM DNQX).

MEAs consisted of a planar array of 60 electrodes (30 μm diameter, 200 μm inter-electrode spacing; Multi Channel Systems, Reutlingen, Germany). Raw electrical signals were amplified, filtered, and digitized through an A/D card (National Instruments, Austin, TX), written to disk and analyzed off-line, as described previously [5]. Retinas were stimulated with a Xenon light source (Sutter Instruments, Novato, CA) fed through a liquid light guide and diffusing filter (Thorlabs Inc., Newton, NJ). Light intensity and wavelength was adjusted with neutral density and narrow band-pass 480 nm interference filters, respectively (Thorlabs, Inc., Newton, NJ). Light intensity was measured with a radiometer (Advanced Photonics International, Fairfield, CT).

### Light exposure protocols

For short-term bleaching/adaptation experiments, retinas were exposed to a 1-min, 480 nm light stimulus at an irradiance (IR) of 3.98 × 10^13^ photon cm^−2^ s^−1^ (log_10_ (3.98 × 10^13^ photon cm^−2^ s^−1^) = IR 13.6) followed by dark recovery intervals of 1 to 10-min followed by a second 1-min test with the same light. Recovery during a test light was calculated as the percentage of spikes during the first 1-min exposure. Age-specific response dynamics were assessed from the first light exposure of each retina in the 1-min exposure experiment. For long-term bleaching/adaptation experiments, retinas were exposed to a continuous, 1-h, 480 nm light stimulus at IR 13.6 and allowed to recover in darkness for 1-h. Activity over the 1-h exposure was monitored continuously and analyzed in 1-min blocks taken every 5-min. To assess recovery, beginning 10-min into the recovery period and every 10-min thereafter, a 1-min test stimulus identical to the exposure light was administered.

For retinoid supplementation experiments, 9-*cis-*retinal (Sigma, St. Louis, MO) was dissolved in acetonitrile, aliquoted and dried under argon for storage at −70 **°**C. For each experiment 9-*cis-*retinal was freshly resuspended in ethanol. Perfusion of retinas was performed as described previously [24]. Briefly, retinas were superperfused for 25 to 30-min with 9-*cis*-retinal in a non-carbogenated HEPES/bicarbonate-buffered physiologic solution (10 mM HEPES, pH 7.4, 125 mM NaCl, 2.5 mM KCl, 1 mM MgCl_2_, 1.25 mM NaH_2_PO_4_, 12 mM glucose, 26 mM NaHCO_3_, 2 mM CaCl_2_, 500 μM glutamine) with 1 % ethanol as a carrier, that was mixed 1:1 with carbogen bubbled bicarbonate-buffered physiologic solution just prior to tissue bath delivery. Retinas were then returned to purely bicarbonate-buffered physiologic solution with glutamatergic blockers for 10-min and tested for light evoked activity.

### ipRGC electrophysiologic subtypes

Categorizing ipRGC subtypes was done as previously described [5] with modifications to reduce light exposure prior to recovery experiments. Subtyping measurements were made at the beginning of each experiment. P8 cells were first divided into cells with on-latencies (time from light-on to time of peak firing) of less than 12-s and greater than 12-s in response to a 1-min, 480 nm light at an irradiance of IR 12.0. Cells with on-latencies less than 12-s were designated Type III cells. Cells with on-latencies greater than 12-s were further divided. Those with an IR 12.0 light response greater than 10 % of their IR 13.6 light responses were designated Type I cells. Those cells with an IR 12.0 light response of less than or equal to 10 % of their IR 13.6 response were categorized as Type II cells. In older animals, cells with on-latencies of less than 12-s in response to a 1-min, 480 nm light at IR 13.0 were designated Type III cells, while those with on-latencies greater than 12-s were Type II cells (Table 4).

### Patch recordings

For single-cell patch physiology experiments, P8 retinas were perfused 7 mls min^−1^ with Ames’ medium (Sigma, St. Louis, MO) containing glutamatergic (50 μM d-AP5, 20 μM d-AP4, and 10 μM DNQX) and cholinergic (5 nM epibatidine) blockers. Light stimuli were delivered using full-field illumination with the blue LED of a LightCrafter 4500 digital light processing projector (Texas Instruments) focused through a 60× water-immersion objective. The calibrated light intensity from the objective was corrected for the blue LED spectrum’s activation of melanopsin to obtain an equivalent 480 nm intensity.

ON-S alpha-like RGCs were targeted for recording in a flat-mount retina preparation based on soma size (> ~15 μm diameter) and shape. Spike responses were recorded using a loose-patch configuration with extracellular solution in the patch pipette. A test flash at IR 12.6 was used to confirm a melanopsin-driven response before proceeding to 1-min light exposures at multiple test light levels. Stimuli were generated and data acquired using open-source software packages Symphony [37] and Stage [38]. Data were analyzed using custom scripts in Matlab (Mathworks, Natick, MA). After spike recording, cells were patched in whole-cell configuration with a cesium-based internal solution and lucifer yellow dye to visualize recorded cells. Retinal mounts were then fixed in 4 % paraformaldehyde and stored in cold phosphate-buffered saline for subsequent immunolabeling and imaging.

### Statistical analysis

Data processing and analysis was performed using custom MATLAB scripts. Statistical analysis was performed with SPSS (IBM, Armonk, NY). Analysis of 1-min and 1-h recovery data used a linear mixed model (LMM) for longitudinal/time sequence datasets. A LMM was used in the 1-min recovery analysis because of missing data (i.e. unresponsive cells) in a repeated measures assessment, which violates ANOVA requirements. A LMM was used in the 1-h recovery analysis because of non-normal data distributions and unbalanced sample sizes (because of the large proportion of Type I cells in P8 recordings). In the LMM fixed effects were age, recovery-time, and age by recovery-time interactions, while the random effect was subject. Because of factor interaction in the 1-min analysis, the LMM was followed by one-way ANOVA and Bonferroni post-hoc tests for individual time point comparisons. In the 1-h recovery, LMM analysis was followed by Bonferroni post hoc tests. When factor interactions were found in the 1-h LMM analysis, individual recovery time points were further analyzed by Kruskal-Wallis (K-W) tests followed by 2-tailed Mann-Whitney (M-W) tests and Bonferroni correction (B-c). When fewer than half of the individual time points were not significant, between group significance is not reported. Analyses of the recovery-time factor are not reported. Non-normal data are plotted in boxplots (whisker plots) with outliers plotted individually if they are greater than 2.7 standard deviations from the mean. Error bars in all other graphs are SEM. Significance is reported at *p* < 0.05 while trends are reported if *p* < 0.06 reported.

## Additional files

Additional file 1:
**Cell density and immune-staining intensity statistics.** Cell density and immuno-staining analysis. Bonferroni corrected. Additional file 2:
**1-min light response dynamics statistics.** 1-min light response dynamics analysis. Kruskal-Wallis (K-W), MannWhitney (M-W), Bonferroni corrected (B-c). Additional file 3:
**1-min light recovery statistics.** 1-min light recovery statistical analysis. Linear mix model (LMM), Bonferroni corrected (B-c). Additional file 4:
**Age specific ipRGC recovery figures.** Recovery of light response parameters following 1-h bright light exposure in P8 (*n* = 60), P15 (*n* = 23), and P30 (*n* = 17) ipRGCs. Additional file 5:
**1-h light recovery by age statistics.** 1-h light recovery statistical analysis by age. Linear mix model (LMM), Kruskal-Wallis (K-W), Mann-Whitney (M-W), Bonferroni corrected (B-c). Additional file 6:
**Age and Subtype specific ipRGC recovery figures.** Recovery of light response parameters following 1-h light exposure in P8 Types I (*n* = 40), II (*n* = 13) and III (*n* = 7) and Post-eye-opening Types II (*n* = 13) and III (*n* = 12) ipRGCs. Additional file 7:
**1-h light recovery statistical analysis by P8 subtypes.** Linear mix model (LMM), Kruskal-Wallis (K-W), Mann-Whitney (M-W), Bonferroni corrected (Bc). Additional file 8:
**1-h light recovery statistical analysis by P15/30 post-eye-opening subtypes.** Linear mix model (LMM), Mann-Whitney (M-W), Bonferroni corrected (B-c).

## Abbreviations

ipRGC: Intrinsically sensitive retinal ganglion cells
P8: Post-natal day 8
P15: Post-natal day 15
P30: Post-natal day 30
P150: Post-natal day 150
MEA: Multielectrode array
IR: Irradiance
d-AP4: d(−)-2-amino-4-phosphonobutyric acid
d-AP5: d(2)-2-amino-5-phosphonopentanoic acid
DNQX: 6,7-dinitroquinoxaline-2,3-dione
SMI-32: Phosphorylated neurofilamant h
IPL: Interplexiform layer
POE: Post-eye-opening
LMM: Linear mixed model
K-W: Kruskal-Wallis test
M-W: Mann-Whitney test
B-c: Bonferroni corrected
