# Supplementary material for: Type I intrinsically photosensitive retinal ganglion cells of early post-natal development correspond to the M4 subtype
Source: Neural Dev. 2015 Jun 21;10:17. doi: 10.1186/s13064-015-0042-x (PMC4480886; doi:10.1186/s13064-015-0042-x)
Supplement: Additional file 1: — Cell density and immune-staining intensity statistics. Cell density and immuno-staining analysis. Bonferroni corrected. [file 13064_2015_42_MOESM1_ESM.pdf]

Additional file 1. Cell density and immune-staining intensity statistics

**Total melanopsin+ density**	**ANOVA, F(2, 45) = 8.7, *p* = 0.001, B-c, P8-P15: *p* = 0.025, P8-P30: p=0.001**
**Melanopsin+/SMI- density**	**ANOVA, F(2, 45) = 0.9, *p* = 0.4**
**Melanopsin+/SMI+ density**	**ANOVA, F(2, 45) = 23.2, *p* = 1.2 x 10^{-7}, B-c, P8-P15: *p* = 0.0003, P15-P30: *p* = 0.056**
**SMI-32 + density**	**ANOVA, F(2, 45) = 4.9, *p* = 0.012, B-c, P8-P15: *p* = 0.061, P15-P30: *p* = 1.0, P8-P30: *p* = 0.015**
**Percent SMI-32 cells also melanopsin+**	**ANOVA F(2, 45) = 26.9, *p* = 1.9 x 10^{-8}, B-c, P8-P15: *p* = 0.021, P15-P30: *p* = 1.7 x 10^{-4}, P8-P30: *p* = 1.2 x 10^{-8}**
**M1 somata intensity**	**K-W, *p* = 0.95**
**M1 dendritic intensity**	**K-W, p = 0.024; M-N, B-c, P8-P15: *p* = 0.058, P15-P30: *p* = 0.050**
**M2 somata intensity**	**K-W, *p* = 0.17**
**M2 dendritic intensity**	**K-W, p = 0.001; M-N, B-c, P8-P15 *p* = 0.009, P15-P30 *p* = 0.002**
**M4 somata intensity**	**K-W, p = 1.9 x 10^{-10}; M-N, B-c, P8-P15: *p* = 1.0 x 10^{-7}, P15-P30: P= 9.6 x 10^{-9}**
**M4 dendritic intensity**	**K-W, p = 6.7 x 10^{-12}; M-N, B-c, P8-P15: *p* = 6.9 x 10^{-9}, P15-P30: *p* = 8.4 x 10^{-9}**

Additional file 1: Cell density and immuno-staining analysis. Bonferroni corrected (B-c).
