# Supplementary material for: Type I intrinsically photosensitive retinal ganglion cells of early post-natal development correspond to the M4 subtype
Source: Neural Dev. 2015 Jun 21;10:17. doi: 10.1186/s13064-015-0042-x (PMC4480886; doi:10.1186/s13064-015-0042-x)
Supplement: Additional file 2: — 1-min light response dynamics statistics. 1-min light response dynamics analysis. Kruskal-Wallis (K-W), MannWhitney (M-W), Bonferroni corrected (B-c). [file 13064_2015_42_MOESM2_ESM.pdf]

Additional file 2. 1-min light response dynamics statistics

**On-latency**	**K-W, *p* = 0.001; M-W, B-c, P8-P15: *p* = 0.006, P8-P30: *p* = 0.012**
**Off-latency**	**K-W, *p* = 0.00005; M-W, B-c, P8-P15: *p* = 0.0002; P15-P30: *p* = 0.012**
**Peak Firing**	**K-W, *p* = 0.007; M-W, B-c, *p* = 0.003**
**Total Spikes**	**K-W, *p* = 0.001; M-W, B-c, P8-P15: *p* = 0.036, P15-P30: *p* = 0.065, P8-P30: *p* = 0.006**

Additional file 2: 1-min light response dynamics analysis. Kruskal-Wallis (K-W), Mann-Whitney (M-W), Bonferroni corrected (B-c).
