# Supplementary material for: Type I intrinsically photosensitive retinal ganglion cells of early post-natal development correspond to the M4 subtype
Source: Neural Dev. 2015 Jun 21;10:17. doi: 10.1186/s13064-015-0042-x (PMC4480886; doi:10.1186/s13064-015-0042-x)
Supplement: Additional file 3: — 1-min light recovery statistics. 1-min light recovery statistical analysis. Linear mix model (LMM), Bonferroni corrected (B-c). [file 13064_2015_42_MOESM3_ESM.pdf]

Additional file 3. 1-min light recovery statistics

**LMM**	**F(numerator df = 12, denominator df = 402.5) = 10.2, Group by Time, $p = 1.4 \times 10^{-17}$**
**1-way ANOVA**	
**1-min:**	**F(2, 77) = 56.7, $p = 7.9 \times 10^{-16}$, B-c, P8-P15: $p = 1.0 \times 10^{-14}$, P8-P30: $p = 1.9 \times 10^{-7}$**
**2-min:**	**F(2, 74) = 42.7, $p = 4.6 \times 10^{-13}$, B-c, P8-P15: $p = 2.6 \times 10^{-9}$, P8-P30: $p = 1.7 \times 10^{-9}$**
**3-min:**	**F(2, 96) = 15.6, $p = 1.3 \times 10^{-6}$, B-c, P8-P15: $p = 0.001$, P8-P30: $p = 1.9 \times 10^{-5}$**
**4-min:**	**F(2, 72) = 13.9, $p = 8.2 \times 10^{-6}$, B-c, P8-P15: $p = 0.008$, P8-P30: $p = 3.8 \times 10^{-5}$**
**5-min:**	**F(2, 76) = 14.1, $p = 6.4 \times 10^{-6}$, B-c, P8-P30: $p = 4.8 \times 10^{-6}$**
**6-min:**	**F(2, 69) = 9.6, $p = 0.0002$, B-c, P8-P30: $p = 0.0001$**

Additional file 3: 1-min light recovery statistical analysis. Linear mix model (LMM), Bonferroni corrected (B-c).
