# Supplementary material for: Type I intrinsically photosensitive retinal ganglion cells of early post-natal development correspond to the M4 subtype
Source: Neural Dev. 2015 Jun 21;10:17. doi: 10.1186/s13064-015-0042-x (PMC4480886; doi:10.1186/s13064-015-0042-x)
Supplement: Additional file 4: — Age specific ipRGC recovery figures. Recovery of light response parameters following 1-h bright light exposure in P8 (n = 60), P15 (n = 23), and P30 (n = 17) ipRGCs. [file 13064_2015_42_MOESM4_ESM.pdf]

Additional file 4: : Age specific ipRGC recovery figures

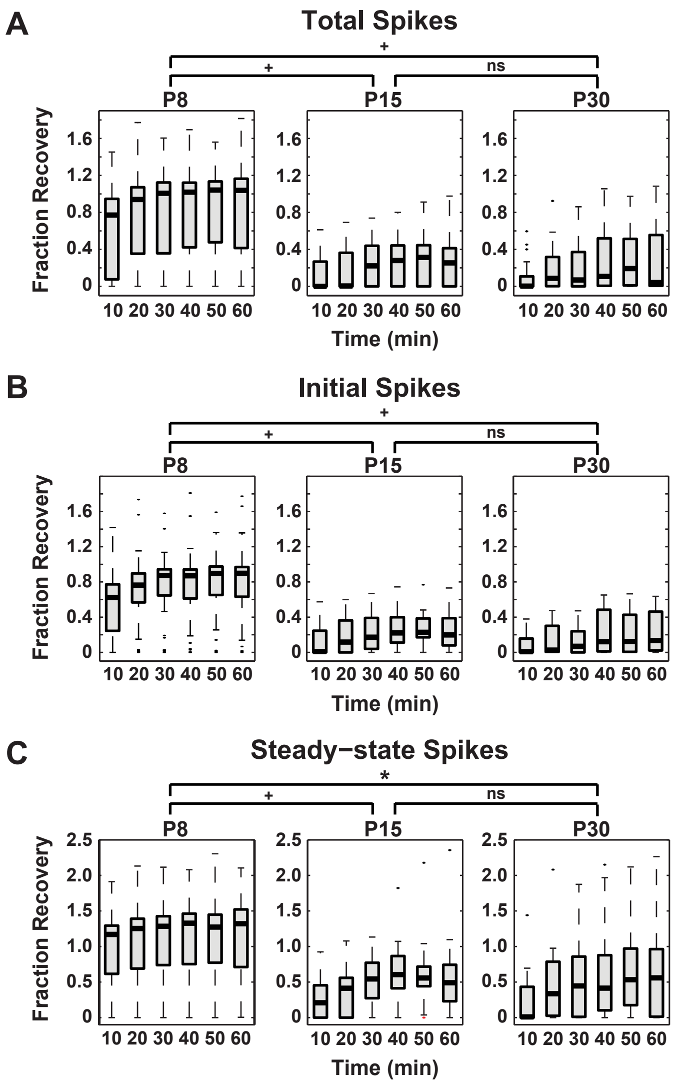

Additional file 4: : Age specific ipRGC recovery figures

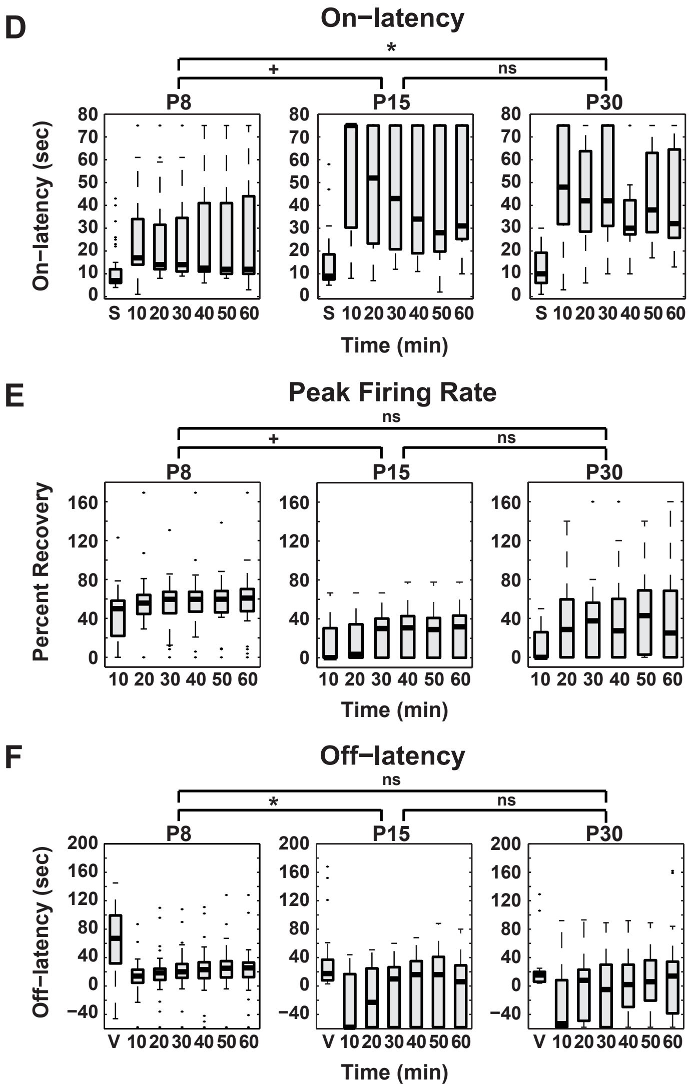

Additional file 4: Recovery of light response parameters following 1-hr bright light exposure in P8 (n=60), P15 (n=23), and P30 (n=17) ipRGCs. Recovery calculated as percent value at beginning of 1-hr light exposure. A) Total spikes, B) Initial spikes (spikes first 30-sec of the 1-min test exposure), and C) Steady-state spikes (spikes from last 30-sec of the 1-min test exposure). D) on-latency (S is the on-latency for the same cells from the start of the 1-hr light exposure), E) peak firing recovery calculated as percent value at beginning of 1-hr light exposure. and F) off-latency (V is the off-latency distribution and median from the 1-min test exposures from Figure 1) No off-latency measurements were made at the beginning of the 1-hr exposure. *: $p < 0.05$, +: $p < 0.005$. LMM followed by either Bonferroni post hoc or Kruskal-Wallis and Mann-Whitney tests with Bonferroni correction.
