# Supplementary material for: Type I intrinsically photosensitive retinal ganglion cells of early post-natal development correspond to the M4 subtype
Source: Neural Dev. 2015 Jun 21;10:17. doi: 10.1186/s13064-015-0042-x (PMC4480886; doi:10.1186/s13064-015-0042-x)
Supplement: Additional file 5: — 1-h light recovery by age statistics. 1-h light recovery statistical analysis by age. Linear mix model (LMM), Kruskal-Wallis (K-W), Mann-Whitney (M-W), Bonferroni corrected (B-c). [file 13064_2015_42_MOESM5_ESM.pdf]

Additional file 5. 1-hr light recovery by age statistics

**On-latency**	**LMM, F(2, 97) = 9.05, p = 2.5 x 10^{-4}, Age, B-c, P8-P15: p = 0.001, P8-P30: p = 0.012**
**Peak Firing**	**LMM, F(10, 485) 3.4, p = 2.3 x 10^{-4}, Age by Time, K-W, p = 1.7 x 10^{-6} to 1.4 x 10^{-4}; M-W, B-c, P8-P15: p = 6.9 x 10^{-6} to 8.7 x 10^{-5}**
**Off-latency**	**LMM, F(2, 97) = 3.5, p = 0.036, Age, B-c, P8-P15: p = 0.039**
**Total Spikes**	**LMM, F(2, 97) = 21.9, p = 1.4 x 10^{-8}, Age, B-c, P8-P15: p = 6.4 x 10^{-7}, P8-P30: p = 1.5 x 10^{-5}**
**Steady-state Spikes**	**LMM, F(16, 485) = 1.9, p = 0.046, Age by Time, K-W, p = 7.0 x 10^{-7} to 2.0 x 10^{-4}; M-W, B-c, P8-P15: p = 2.0 x 10^{-5} to 3.3 x 10^{-4}, P8-P30: p = 0.0001 to 0.024, 50-min not-significant**
**Initial Spikes**	**LMM, F(10, 485) = 2.5, p = 0.005, Age by Time, K-W, p = 1.0 x 10^{-7} to 9.3 x 10^{-7}; M-W, B-c, P8-P15: p = 4.8 x 10^{-6} to 2.0 x 10^{-5}, P8-P30: p = 6.0 x 10^{-5} to 3.0 x 10^{-4}**

Additional file 5: 1-hr light recovery statistical analysis by age. Linear mix model (LMM), Kruskal-Wallis (K-W), Mann-Whitney (M-W), Bonferroni corrected (B-c).
