# Supplementary material for: Type I intrinsically photosensitive retinal ganglion cells of early post-natal development correspond to the M4 subtype
Source: Neural Dev. 2015 Jun 21;10:17. doi: 10.1186/s13064-015-0042-x (PMC4480886; doi:10.1186/s13064-015-0042-x)
Supplement: Additional file 6: — Age and Subtype specific ipRGC recovery figures. Recovery of light response parameters following 1-h light exposure in P8 Types I (n = 40), II (n = 13) and III (n = 7) and Post-eye-opening Types II (n = 13) and III (n = 12) ipRGCs. [file 13064_2015_42_MOESM6_ESM.pdf]

Additional file 6: Age and Subtype specific ipRGC recovery figures

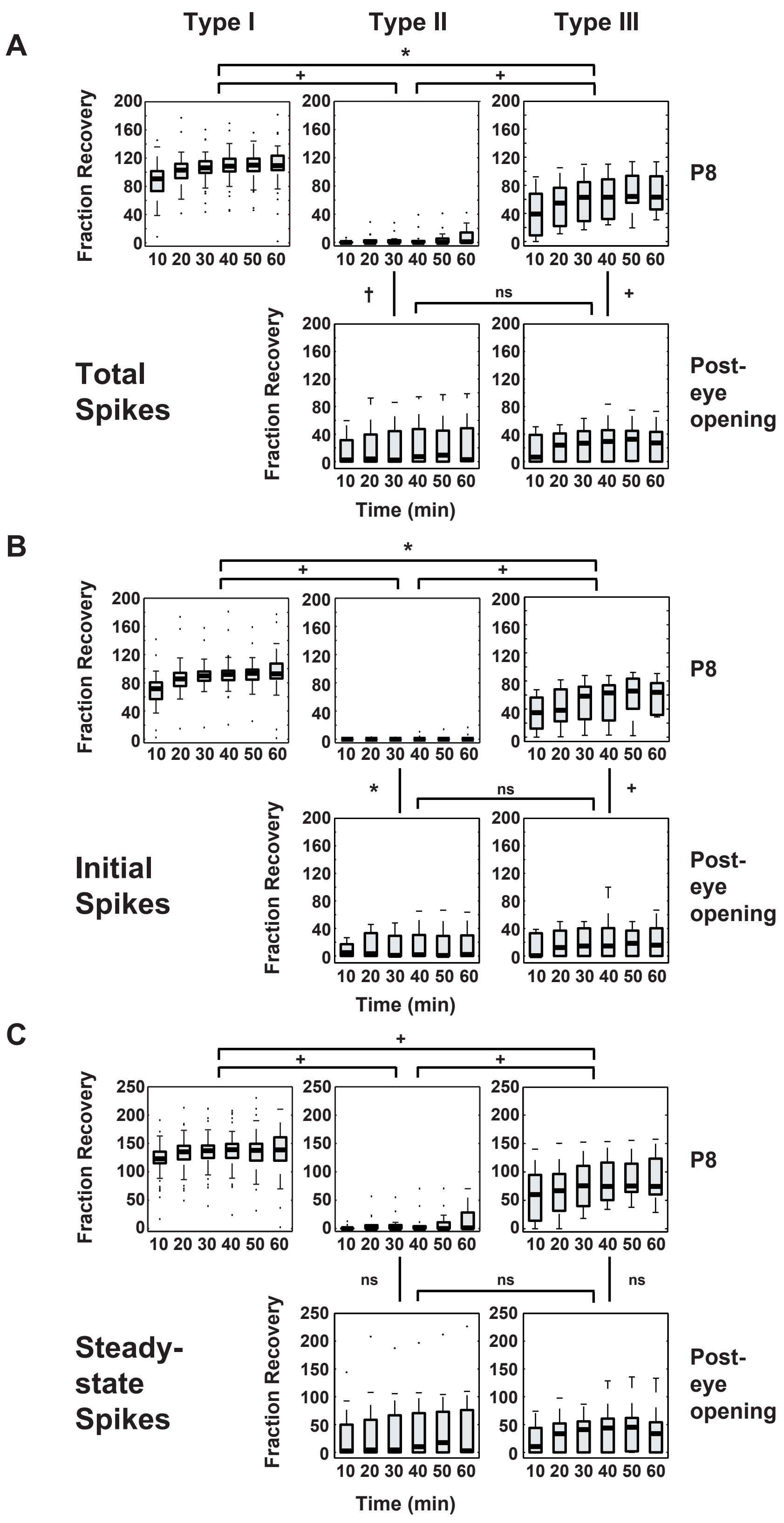

Additional file 6: Age and Subtype specific ipRGC recovery figures

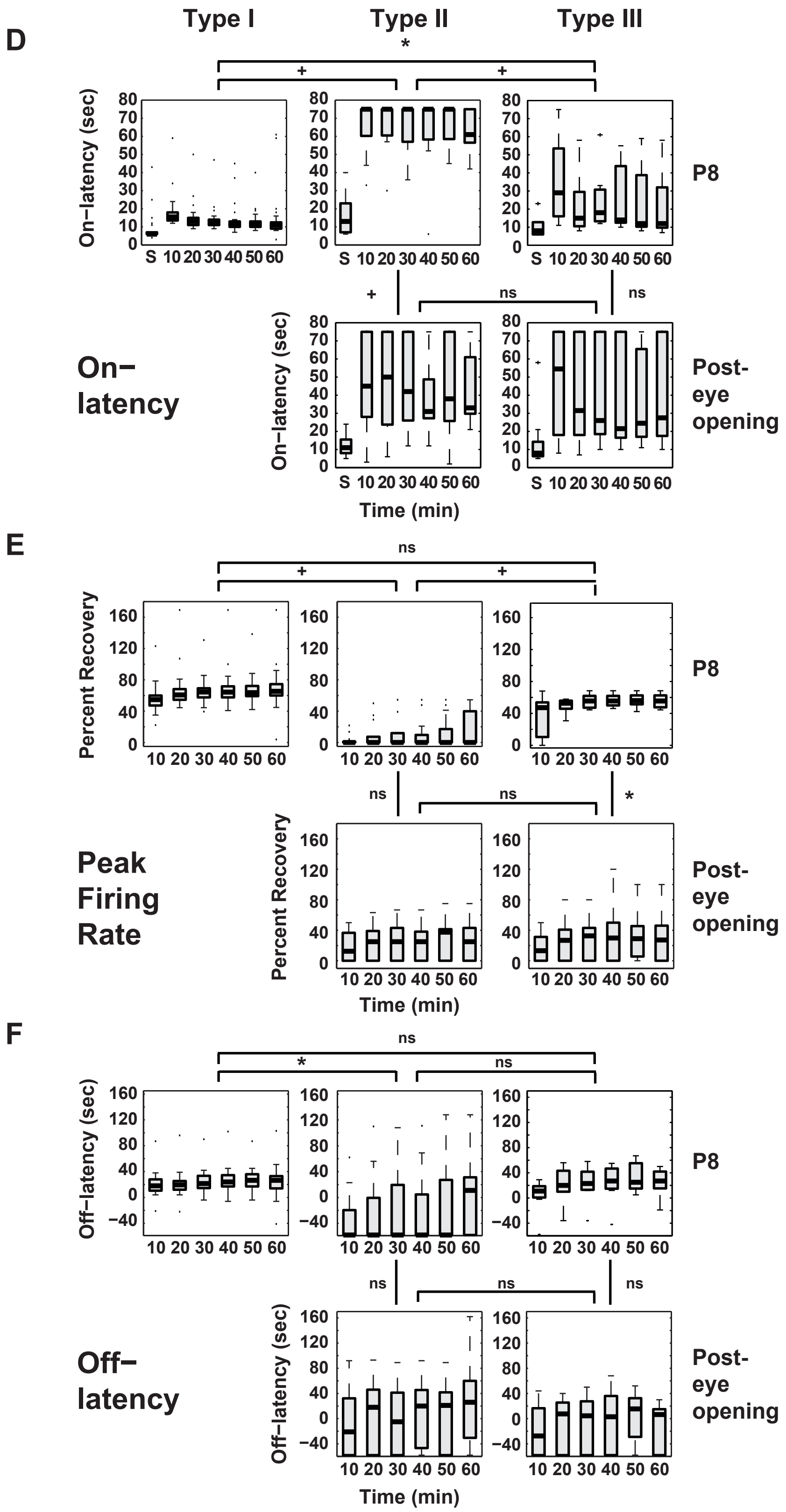

Additional file 6: Recovery of light response parameters following 1-hr light exposure in P8 Types I (n=40), II (n=13) and III (n=7) and Post-eye-opening Types II (n=13) and III (n=12) ipRGCs. Recovery calculated as percent value at beginning of 1-hr light exposure. A) Total Spikes. B) Initial spikes. C) Steady-state spikes, D) On-latency (S is the on-latency for the same cells from the start of the 1-hr light exposure). E) Peak firing, and F) off-latency. *: $p < 0.05$, +: $p < 0.005$, † $p = 0.05$. LMM followed by either Bonferroni post hoc or Kruskal-Wallis and Mann-Whitney tests with Bonferonni correction.
