# Supplementary material for: Type I intrinsically photosensitive retinal ganglion cells of early post-natal development correspond to the M4 subtype
Source: Neural Dev. 2015 Jun 21;10:17. doi: 10.1186/s13064-015-0042-x (PMC4480886; doi:10.1186/s13064-015-0042-x)
Supplement: Additional file 7: — 1-h light recovery statistical analysis by P8 subtypes. Linear mix model (LMM), Kruskal-Wallis (K-W), Mann-Whitney (M-W), Bonferroni corrected (Bc). [file 13064_2015_42_MOESM7_ESM.pdf]

Additional file 7. 1-hr recovery of P8 subtypes statistics

**On-Latency**	**LMM, F(2, 57) = 152.2, p = 1.4 x 10^{-23}, Type, B-c, Type I-II: p = 4.7 x 10^{-24}, Type I-III: p = 0.01, Type II-III: p = 1.4 x 10^{-23}**
**Peak Firing**	**LMM, F(2, 57) = 53.8, p = 7.5 x 10^{-14}, Type, B-c, Type I-II: p = 2.9 x 10^{-14}, Type I-III: p = 3.6 x 10^{-6}**
**Off-Latency**	**LMM, F(2, 285) = 5.7, p = 8.7 x 10^{-8}, Type by Time, K-W, p = 5.6 x 10^{-5} to 0.025; M-W, B-c, Type I-II: p = 2.0 x 10^{-5} to 0.024, 60-min not-significant**
**Total Spikes**	**LMM, = F(10, 285) = 2.3, p = 0.01, Type by Time, K-W, p = 1.7 x 10^{-13} to 2.3 x 10^{-12}; M-W, B-c, Type I-II: p = 1.9 x 10^{-11} to 9.6 x 10^{-11}, Type II-III: p = 0.01 to 0.02 (10-20- and 60-min not significant), Type I-III: p = 2.2 x 10^{-8} to 1.5 x 10^{-7}**
**Initial Spikes**	**LMM, F(10, 283) = 5.2, p = 4.8 x 10^{-7}, Type by Time, K-W, p = 4.5 x 10^{-15} to 4.6 x 10^{-14}; M-W, B-c, Type I-II: p = 3.0 x 10^{-11} to 4.2 x 10^{-10}, Type II-III: p = 0.003 to 0.004 (10-min not significant), Type I-III: p = 5.4 x 10^{-8} to 1.5 x 10^{-6}**
**Steady-State Spikes**	**LMM, F(2, 57) = 71.8, P= 2.65 x 10^{-16}, Type, B-c, Type I-II: p = 1.6 x 10^{-16}, Type I-III: p = 8.4 x 10^{-5}, Type II-III: p = 0.001**

Additional file 7: 1-hr light recovery statistical analysis by P8 subtypes. Linear mix model (LMM), Kruskal-Wallis (K-W), Mann-Whitney (M-W), Bonferroni corrected (B-c).
