# Supplementary material for: Type I intrinsically photosensitive retinal ganglion cells of early post-natal development correspond to the M4 subtype
Source: Neural Dev. 2015 Jun 21;10:17. doi: 10.1186/s13064-015-0042-x (PMC4480886; doi:10.1186/s13064-015-0042-x)
Supplement: Additional file 8: — 1-h light recovery statistical analysis by P15/30 post-eye-opening subtypes. Linear mix model (LMM), Mann-Whitney (M-W), Bonferroni corrected (B-c). [file 13064_2015_42_MOESM8_ESM.pdf]

Additional file 8. 1-hr recovery of post-eye-opening (P15/P30) subtypes statistics

**Type II**	
**On-latency**	**LMM, F(2, 24) 12.2, *p* = 0.002, Age, B-c, P8-post-eye-opening:*p* = 0.002**
**Total Spikes**	**LMM, F(2, 24) = 4.3, *p* = 0.050, Age, B-c, P8-post-eye-opening: *p* = 0.050**
**Initial Spikes**	**LMM, F(2, 24) = 6.7, *p* = 0.02, Age, B-c, P8-post-eye-opening: *p* = 0.02**
**Type III**	
**Peak Firing**	**LMM, F(1, 17) = 6.4, *p* = 0.03, Age, B-c, P8-post-eye-opening: *p* = 0.03**
**Total Spikes**	**LMM, F(1, 17) = 8.7, P= 0.009, Age, B-c, P8-post-eye-opening: *p* = 0.009**
**Initial Spikes**	**LMM, F(5, 83.9) = 2.5, *p* = 0.036, Age by Time; M-W, B-c, P8-post-eye-opening: $p = 1.4 \times 10^{-5}$ to 6.0×10^{-5}**

Additional file 8: 1-hr light recovery statistical analysis by P15/30 post-eye-opening subtypes. Linear mix model (LMM), Mann-Whitney (M-W), Bonferroni corrected (B-c).
